# The Role of Humor and Self-Care in Longevity: A Systematic Review of Psychological and Behavioral Health Literature

**DOI:** 10.7759/cureus.101820

**Published:** 2026-01-19

**Authors:** Aneta Toti, Zoe Konstanti, Konstantinos Baliotis, Stefanos Mantzoukas, Mary Gouva, Michael Kourakos

**Affiliations:** 1 Research Laboratory Psychology of Patients, Families and Health Professionals, Department of Nursing, School of Health Sciences, University of Ioannina, Ioannina, GRC

**Keywords:** longevity, mental health, psychological resilience, quality of life, well-being

## Abstract

A complex interplay between behavioral, environmental, and genetic factors influences longevity. According to recent studies, psychological determinants, humor and self-care in particular, are crucial for fostering health and prolonging life. Evidence about the function of humor and self-care as psychological coping strategies that may promote longevity is compiled in this systematic review. A thorough search for peer-reviewed research published between January 2000 and March 2024 was conducted in PubMed, PsycINFO, Scopus, and Web of Science. Both qualitative and quantitative studies examining the relationships between humor, self-care behaviors, and longevity-related health outcomes were eligible. After screening 742 records in accordance with the Preferred Reporting Items for Systematic Reviews and Meta-Analyses (PRISMA) guidelines, 16 studies met the inclusion criteria and were included in the qualitative synthesis. Across the included studies, humor was consistently associated with reduced psychological stress, improved psychological well-being, enhanced immune and cardiovascular markers, and, in some longitudinal cohorts, lower mortality risk. Self-care practices, including mindfulness, physical activity, adequate sleep, and social engagement, were associated with reduced allostatic load, greater psychological resilience, and better functional and cardiometabolic health. These psychological elements may work in concert to promote healthy aging trajectories by acting as protective barriers against long-term stress and age-related decline. This review emphasizes the promise of self-care and humor as accessible, low-cost strategies for improving well-being and supporting healthy aging; however, further longitudinal and experimental research is required to establish causality and clarify underlying biological and psychological mechanisms.

## Introduction and background

Human lifespan is influenced by a variety of factors, such as lifestyle choices, psychological health, environmental circumstances, and genetic composition [1]. Research has historically concentrated on physical health determinants, including access to healthcare, physical activity, and nutrition, but there is growing awareness of the importance that psychological and emotional factors play in determining lifespan and quality of life [2]. Adaptive coping mechanisms, emotional resilience, and efficient stress management have all drawn more attention recently due to their positive effects on longevity and general health [3].

Self-care and humor are becoming prominent research topics among these psychological aspects [4]. Both comedy and self-care, which were once thought to be secondary to physical health practices, are now acknowledged for their positive physiological and psychological effects [5]. For instance, humor is known to be a powerful stress reduction technique. It has been associated with greater social connectedness, a stronger immune system, a decreased sense of pain, and, in certain research, a longer life expectancy [6]. Consistent self-care behaviors, like mindfulness, getting enough sleep, eating a healthy diet, expressing emotions, and looking for social support, are also becoming more widely recognized as being essential for maintaining mental health and preventing chronic illnesses [7].

According to recent studies, self-care and comedy may have preventive benefits that go beyond temporarily elevating mood [8]. The risk of age-related diseases and mortality may be decreased by these behaviors, which may help minimize biological wear and tear, also known as allostatic load, by reducing stress and encouraging pleasant mental states [9,10].

However, despite growing interest, evidence on the combined and independent roles of humor and self-care in promoting longevity remains fragmented, and no comprehensive synthesis has systematically examined their associations with longevity-related outcomes across study designs and populations.

This systematic review aims to synthesize and critically evaluate existing psychological and behavioral health research on humor and self-care in relation to longevity-related outcomes. Specifically, the review seeks to clarify the role of these psychological resources in supporting healthy aging and to explore their potential integration into public health strategies and aging interventions.

## Review

Methods

Search Strategy

Four electronic databases, namely, PubMed, PsycINFO, Scopus, and Web of Science, were used to do a systematic search. The search terms included "humor", "self-care", "longevity", "lifespan", "coping", "resilience", and "mental health". The review investigated studies that were published between 2000 and 2024, and only articles that were written in English were used (Table 1).

**Table 1:** Electronic database search strategy This table summarizes the electronic databases searched, key search terms, publication date range, and filters applied to identify studies examining humor, self-care practices, and longevity-related outcomes. Searches were conducted in accordance with PRISMA guidelines. PRISMA: Preferred Reporting Items for Systematic Reviews and Meta-Analyses

Database	Search terms	Date range	Filters
PubMed	("humor" OR "laughter") AND ("self-care" OR "mindfulness") AND ("longevity" OR "aging")	2000-2024	English, humans
PsycINFO	humor AND self-care AND aging	2000-2024	Peer-reviewed
Scopus	humor AND longevity	2000-2024	Articles
Web of Science	humor AND health AND aging	2000-2024	English

Eligibility Criteria

The eligibility criteria for this review were developed using the PICO (Population, Intervention/Exposure, Comparison, and Outcomes) framework to ensure a focused and systematic selection of studies (Table 2). The population of interest included adults aged 60 years and older from any geographical or cultural background. Interventions were defined as exposure to humor-related activities or self-care practices, such as laughter therapy, mindfulness, or other lifestyle behaviors promoting health and psychological well-being. Comparisons were not restricted; studies with or without explicit comparison groups were included, provided they explored associations between humor or self-care and health outcomes. Outcomes of interest encompassed measures related to longevity, including lifespan, health span, and health-related biomarkers such as stress levels, immune function, cardiovascular parameters, and mortality rates. Studies were included if they were peer-reviewed empirical research, quantitative, qualitative, or mixed methods, that examined humor and/or self-care in relation to these outcomes.

**Table 2:** PICO framework used in this review This table outlines the PICO criteria used to define study eligibility and guide the systematic selection of literature for this review. PICO: Population, Intervention/Exposure, Comparison, and Outcomes

PICO component	Description
Population (P)	Older adults (generally ≥60 years), including advanced-age populations where applicable
Intervention/Exposure (I)	Humor-related activities (e.g., laughter, humor styles, humor-based interventions) and self-care practices (e.g., mindfulness, physical activity, sleep hygiene)
Comparison (C)	Lower exposure, usual care, or no explicit comparison
Outcomes (O)	Longevity, mortality, health span, functional health, psychological well-being, stress-related biomarkers

Inclusion Criteria

Only peer-reviewed empirical studies were included. Eligible studies involved older adult populations (generally aged ≥60 years), including advanced-age and centenarian populations where applicable. Studies were required to examine humor and/or self-care in relation to longevity or health-related outcomes and to report quantitative or qualitative data on mortality, health span, functional health, or relevant health biomarkers.

Exclusion Criteria

Studies that were not based on primary data (such as opinion articles, editorials, or commentaries), studies that only looked at children or teenagers, and studies that looked at humor as entertainment without considering longevity or health were also excluded. Excluded were studies that only addressed self-care in acute medical settings without mentioning long-term health consequences.

Data Extraction and Synthesis

This systematic review was conducted in accordance with the PRISMA guidelines. During data extraction, key information, including study characteristics, participant demographics, exposure or intervention details, and primary findings, was recorded. A thematic synthesis approach was used to synthesize findings across studies.

Risk of bias was assessed for all empirical studies included in the qualitative and quantitative synthesis using design-appropriate validated tools (Table 3). Observational cohort and cross-sectional studies were assessed using the Newcastle-Ottawa Scale (NOS), randomized controlled trials were evaluated using the Cochrane Risk of Bias tool, non-randomized interventional studies were assessed using the ROBINS-I (Risk Of Bias In Non-randomized Studies of Interventions) tool, and qualitative studies were appraised using the Critical Appraisal Skills Programme (CASP) qualitative checklist [11-14]. Risk-of-bias assessments were conducted at the study level and summarized narratively and in tabular form. One included study was not subjected to a formal risk-of-bias assessment because it did not involve empirical outcome evaluation relevant to longevity and was therefore not eligible for risk-of-bias appraisal.

**Table 3:** Risk-off-bias assessment of the included studies This table summarizes the risk-of-bias assessment for representative studies included in the qualitative synthesis. Risk of bias was evaluated using validated, design-specific tools. NOS: Newcastle–Ottawa Scale; CASP: Critical Appraisal Skills Programme; ROBINS-I: Risk Of Bias In Non-randomized Studies of Interventions

Study	Design	Tool used	Selection/confounding bias	Performance bias	Detection/measurement bias	Overall risk
Koch et al., 2007 [15]	Qualitative interviews	CASP (qualitative)	Low	N/A	Low	Low
Svebak et al., 2010 [16]	Prospective cohort	NOS (cohort)	Low	Low	Moderate	Moderate
Tamada et al., 2021 [17]	Prospective cohort	NOS (cohort)	Low	Low	Low	Low
Funakubo et al., 2024 [18]	Cross-sectional	NOS (cross-sectional)	Moderate	N/A	Moderate	Moderate
Parker et al., 2023 [19]	Observational exploratory	NOS (cross-sectional)	Moderate	N/A	Moderate	Moderate
Gimenez et al., 2021 [20]	Cross-sectional survey	NOS (cross-sectional)	Moderate	N/A	Moderate	Moderate
Kato et al., 2016 [21]	Observational (centenarians)	NOS (cross-sectional)	Moderate	N/A	Low	Moderate
Lima et al., 2012 [22]	Epidemiological survey	NOS (cross-sectional)	Moderate	N/A	Moderate	Moderate
Celso et al., 2003 [23]	Cross-sectional	NOS (cross-sectional)	Moderate	N/A	Moderate	Moderate
Inoue et al., 2022 [24]	Cross-sectional (JAGES)	NOS (cross-sectional)	Low	N/A	Low	Low
Cherry et al., 2018 [25]	Observational	NOS (cohort)	Moderate	Low	Moderate	Moderate
Hirosaki et al., 2013 [26]	Randomized controlled trial	Cochrane RoB	Low	Moderate	Low	Low
Rezaei et al., 2019 [27]	Non-randomized intervention	ROBINS-I	Moderate	Moderate	Moderate	Moderate
Kuru Alıcı et al., 2018 [28]	Non-randomized pilot	ROBINS-I	Moderate	Moderate	Moderate	Moderate
Chamorro-Garrido et al., 2021 [29]	Interventional (non-randomized)	ROBINS-I	Moderate	Moderate	Low	Moderate

Results

Study Selection

There were 852 records found in the first database search across PubMed, Scopus, PsycINFO, and Web of Science. Prior to deduplication, there were 926 documents altogether after an additional 74 were found by hand searching Google Scholar and looking through the reference lists of pertinent papers. After duplicates were eliminated, 742 records were left for screening. For a variety of reasons, including non-comparative designs, a lack of cultural context, a concentration on individuals under 85, a lack of exposure to comedy or self-care, and limited language accessibility, 610 records were eliminated during the title and abstract screening phase. Of the 16 studies included in the qualitative synthesis, four provided sufficient quantitative data and were therefore included in a quantitative synthesis (meta-analysis), while the remaining studies contributed to the narrative qualitative synthesis.

**Figure 1:**
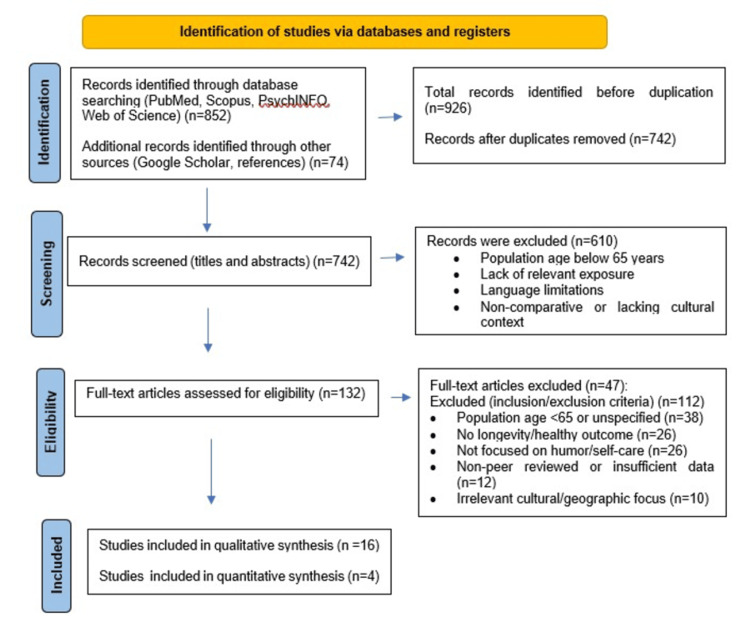
PRISMA flow diagram summarizing the identification, screening, eligibility, and inclusion of studies in the systematic review The diagram summarizes the process of identifying records through database searching and other sources, removing duplicates, screening titles and abstracts, assessing full-text articles for eligibility, and determining the final studies included in the qualitative and quantitative syntheses. PRISMA: Preferred Reporting Items for Systematic Reviews and Meta-Analyses

This process left 132 full-text articles for detailed assessment. The following criteria were used to exclude 112 articles after full-text review: lack of humor or self-care (n=26), study populations >85 years of age or unspecified age (n=38), irrelevant cultural or geographic focus (n=10), non-peer-reviewed publications or insufficient data (n=12), and lack of longevity- or health-related outcomes (n=26) (Figure 1). 

Study Characteristics

The qualitative synthesis included 16 studies with various designs, such as narrative reviews, systematic reviews, cross-sectional studies, and longitudinal observational studies. According to the inclusion criteria of the review, the majority of studies concentrated on older adult populations that were 60 years of age or older. Sample sizes ranged widely, from small community-based samples to large epidemiological cohorts. The studies provided a culturally comprehensive view on humor and self-care practices linked to lifespan since they represented a geographically diverse set of contexts, including North America, Europe, and portions of Asia.

Summary of Findings

There was a constant correlation between humor and better psychological health, less perceived stress, stronger immunity, and, in some situations, a lower chance of death. The psychological advantages of humor seemed to differ depending on the type; for instance, affiliative and self-enhancing humor were more helpful for stress reduction and resilience [4,15,20].

The included research examined a variety of self-care practices, from mindfulness-based stress reduction and emotional regulation to regular exercise and good sleep hygiene [30,31]. These behaviors were often associated with reduced allostatic load, better cardiovascular health, and increased resilience to psychological and physiological stressors [32].

Even while the majority of research found beneficial correlations between humor, self-care, and outcomes related to longevity, the majority of the evidence was correlational [33,34]. Only four of the included studies offered quantitative information appropriate for meta-analysis. Although the experimental designs required to prove causation were absent from the remaining investigations, they nevertheless offered insightful qualitative information (Table 4).

**Table 4:** Characteristics and key findings of the studies included in the qualitative synthesis This table summarizes the authors, study aims and designs, sample characteristics and settings, and principal findings of the studies included in the qualitative synthesis of the systematic review examining humor, laughter, self-care, and longevity-related outcomes in older adults. *Psychometric validation study included as supporting literature; not assessed for the risk of bias. HR: hazard ratio; OT: occupational therapy; RCT: randomized controlled trial; OR: odds ratio

Authors	Aim/design	Sample and setting	Key findings
Koch et al., 2007 [15]	Explore experiences and perceptions of aging	24 centenarians; conversational interviews	Counter ageism, longevity tips: active, simple life, humor; challenges: eyesight loss; resilience via family ties
Svebak et al., 2010 [16]	Test humor-survival link	66,000 adults, Norway; 7-year follow-up	Higher humor → lower mortality (HR 0.50); effect weaker >65 years; independent of gender/health
Funakubo et al., 2024 [18]	Examine laughter, social activity, depression vs. oral frailty	916 adults (60-79), Fukushima	Daily laughter and no depression ↓ oral frailty; multivariate: only daily laughter and no depression significant
Parker et al., 2023 [19]	Explore playfulness vs. well-being	123 adults 60+, Australia	Playfulness ↑ well-being; strongest: other-directed; OT use recommended
Tamada et al., 2021 [17]	Prospective cohort	14,233 adults ≥65, Japan; 3 years	Rare laughter ↑ disability (HR 1.42); no mortality link
Chamorro-Garrido et al., 2021 [29]	Positive psychology intervention	111 institutionalized older adults; 11 weeks	Gains in well-being sustained 12 months; effective simple tool
Gimenez et al., 2021 [20]	Examine happiness vs. subjective life expectancy	1,298 Chilean seniors	Happiness strongest predictor; effect ↑ with age; promoting happiness supports active aging
Cherry et al., 2018 [25]	Examine humor and spirituality in resilience	Disaster survivors (Katrina, Rita, Deepwater Horizon)	Humor and spiritual support ↑ resilience; disrupted work and low income ↓ resilience
Rezaei et al., 2019 [27]	Laughter therapy effect	32 nursing home residents; 1 month	QoL ↑ vs. control; effect lasted 1 month
Kuru Alıcı et al., 2018 [28]	Pilot intervention	50 nursing home elders; 5 weeks	Loneliness ↓; death anxiety ↓; supports use in nursing homes
Kato et al., 2016 [21]	Examine centenarians	54 adults 98-107	Positive attitude and self-rated health ↓ depression; mediates health-depression link
Lima et al., 2012 [22]	Epidemiological survey	1,431 elderly	Happiness ↑ with marriage, activity, diet, sleep, health; low disability and chronic illness best
Celso et al., 2003 [23]	Cross-sectional study	Assisted-living older adults	Better health ↑ humor coping and life satisfaction; humor did not mediate link
Hirosaki et al., 2013 [26]	RCT	≥60 years, Japan; 10 weeks	Improved bone density, HbA1c, self-rated health; increased motivation for activity
Caycho-Rodríguez et al., 2018 [35]*	Psychometric validation	236 Peruvian older adults	One-dimensional, reliable, correlated with life satisfaction and humor
Inoue et al., 2022 [24]	Cross-sectional	19,452 Japanese ≥65	Better vision ↑ laughter (OR 1.72); poor vision ↓ (OR 0.86)

Humor-related health and longevity outcomes: Across the studies summarized in Table 1, humor-related variables were consistently associated with favorable psychological outcomes, including reduced depressive symptoms, enhanced emotional resilience, and improved coping capacity in older adults. Large-scale cohort studies demonstrated associations between higher levels of humor or laughter frequency and reduced mortality risk or functional decline, although effect sizes varied by age group and outcome measure. Interventional studies further suggested that structured laughter or humor-based activities may improve subjective well-being and selected physiological indicators, such as metabolic and cardiovascular parameters [28-30,32,34].

Further evidence for the psychological and practical advantages of comedy came from cross-sectional studies [14,18-20]. In several cultural contexts, frequent laughter was associated with improved oral health outcomes, reduced loneliness, fewer depressive symptoms, and greater subjective well-being [22-24,28]. Crucially, a growing body of research distinguishes between different kinds of humor, demonstrating that self-enhancing and affiliative humor styles are more consistently linked to stress reduction and resilience than aggressive or self-defeating humor styles [4,30,36]. These results imply that not all humor has the same health benefits and that social and adaptive humor may be more important as people age.

Potential causal pathways were proposed by evidence from interventional research, albeit in smaller numbers. Findings from both randomized and non-randomized evaluations of laughter-based therapies indicated benefits for metabolic indicators, self-rated health, quality of life, and motivation for physical activity [5,22-25]. These results suggest that humor-based activities may offer low-cost, scalable approaches to improving psychological and functional health in older adults, although sample sizes were often modest and follow-up periods were brief.

Self-care practices and health outcomes: A wide range of behaviors, including mindfulness, emotional control, exercise, good sleep hygiene, and participation in fulfilling daily activities, were included in studies of self-care practices. Greater engagement in self-care practices was associated with better functional health outcomes, lower perceived stress, and higher psychological resilience across both observational and interventional study designs [32-34,37]. Self-care practices have been repeatedly associated with lower allostatic load and reduced risk of chronic illness in epidemiological research, underscoring their potential importance in extending health rather than lifespan [9,22,38].

Emotional self-care and mindfulness-based therapies were especially linked to better coping skills, better stress management, and decreased depressive symptoms [32-34]. By encouraging better behavioral patterns and lowering long-term physiological stress reactions, these psychological advantages may have an indirect impact on longevity [9]. Regular exercise and enough sleep are examples of behavioral self-care behaviors that have been more closely linked to functional independence and cardiometabolic health markers, underscoring their significance in older populations [38,39].

Notably, the advantages of self-care behaviors emerged across a variety of geographical and cultural contexts, indicating widespread applicability [8,33]. However, direct comparison between studies was hampered by the variability in how self-care was operationalized and measured [34,40]. Despite these drawbacks, the body of research demonstrates that self-care is a multifaceted concept with significant effects on the long-term health and psychological well-being of older individuals [9].

Heterogeneity and null findings: Significant heterogeneity in the literature was highlighted by several studies that returned mixed or null results despite generally positive relationships. Inconsistent results were probably caused by variations in study design, outcome definitions, and measuring techniques. For instance, while some research found links between the frequency of laughter and death, other studies found more connections with psychological well-being or functional deterioration than with survival [16,17].

One major issue is measurement variability. Comparability between trials was limited because humor was assessed using diverse markers, including self-reported laughter frequency, humor style questionnaires, and involvement in structured interventions [18,30,36]. The synthesis was further complicated by the fact that outcomes ranged from biological indicators and subjective well-being to mortality and disability [23]. The protective effects of humor seemed to be reduced in more advanced age groups, suggesting that age-related variations may also be a factor [16,24].

Overall, our results emphasize the need for more standardized assessment techniques and long-term interventional designs to elucidate causal pathways and population-specific effects, even though they do not rule out the possible advantages of humor and self-care.

Discussion

The findings of this review suggest that humor and self-care may play a meaningful role in promoting longevity, not only by enhancing life satisfaction but also by influencing biological, behavioral, and social processes [41,42]. Several mechanisms may explain these associations. From a biological perspective, humor appears to reduce physiological stress responses. Engaging in laughter or lighthearted social interactions has been linked to reductions in cortisol levels, a key marker of stress. The capacity to appraise stressors with humor and to emotionally "let go" of negative events may function as a protective factor, mitigating the harmful effects of chronic stress on the body [36].

Behaviorally, individuals who regularly engage in self-care practices or utilize humor as a coping strategy are often more likely to maintain health-promoting routines, including regular physical activity, balanced nutrition, and adequate sleep [37]. These lifestyle behaviors are well-documented contributors to physical health and longevity [38,39].

Social factors also play an important role. Certain types of humor, especially affiliative or socially bonding humor, are associated with stronger interpersonal relationships and more robust social support networks [43,44]. Social connection itself is a well-established predictor of both physical and mental health outcomes, including reduced mortality risk [7].

In the context of self-care, its role has expanded beyond the prevention of burnout to encompass resilience-building and recovery from stress, illness, or emotional exhaustion [36]. When integrated into daily routines, self-care practices may enhance both psychological resilience and physiological recovery processes [8,45].

Humor and self-care may work together to affect longevity-related outcomes beyond their separate benefits. By promoting social connection, emotional regulation, and cognitive appraisal of stressors, humor can reduce perceived stress and foster positive interpersonal interactions [7,30,36]. These social and psychological mechanisms may, therefore, enhance adherence to self-care practices, including exercise, good sleep hygiene, and mindfulness. However, regular self-care may boost psychological adaptability and emotional reserves, making people more open to using comedy as a coping mechanism [34,40]. This reciprocal relationship suggests that humor and self-care are interrelated elements within broader frameworks of resilience and healthy aging, rather than separate protective factors.

Practically speaking, the review's conclusions encourage the use of self-care and humor-based tactics in aging and health promotion initiatives. Because humor-oriented interventions are inexpensive, low-risk, and culturally flexible, they are especially well-suited for community and institutional settings, such as assisted living facilities and nursing homes [5,26,28]. Examples of these interventions include structured laughter exercises or playful social engagement. In a similar vein, self-care programs that prioritize emotional control, mindfulness, and regular health-promoting activities may support older persons in preserving their psychological well-being and functional independence [33,40]. Even in extremely old age, including these strategies in geriatric care models and preventive public health campaigns may help prolong life and improve quality of life [7].

However, important limitations in the current literature must be acknowledged. Much of the existing research relies on self-reported behaviors, which can introduce recall and reporting biases. Additionally, there is considerable variability in study design and measurement approaches across the studies reviewed. Notably, most findings are correlational in nature, limiting the ability to draw causal conclusions about the effects of humor and self-care on longevity. To better understand these relationships, future research should prioritize longitudinal designs and controlled interventions that can more clearly establish temporal and causal links [46,47].

## Conclusions

Self-care and humor are two potential psychological techniques that can help people live longer and be healthier overall. Their potential to improve physical and mental well-being throughout life is supported by an expanding corpus of research that offers a growing empirical and theoretical base. Although the research now available suggests a robust correlation between humor, self-care, and better health outcomes, the underlying causative pathways are still not fully understood.
